# Human Retroviral Host Restriction Factors APOBEC3G and APOBEC3F Localize to mRNA Processing Bodies

**DOI:** 10.1371/journal.ppat.0020041

**Published:** 2006-05-12

**Authors:** Michael J Wichroski, G. Brett Robb, Tariq M Rana

**Affiliations:** Department of Biochemistry and Molecular Pharmacology, University of Massachusetts Medical School, Worcester, Massachusetts, United States of America; The Salk Institute for Biological Studies, United States of America

## Abstract

APOBEC3G is an antiviral host factor capable of inhibiting the replication of both exogenous and endogenous retroviruses as well as hepatitis B, a DNA virus that replicates through an RNA intermediate. To gain insight into the mechanism whereby APOBEC3G restricts retroviral replication, we investigated the subcellular localization of the protein. Herein, we report that APOBEC3G localizes to mRNA processing (P) bodies, cytoplasmic compartments involved in the degradation and storage of nontranslating mRNAs. Biochemical analysis revealed that APOBEC3G localizes to a ribonucleoprotein complex with other P-body proteins which have established roles in cap-dependent translation (eIF4E and eIF4E-T), translation suppression (RCK/p54), RNA interference–mediated post-transcriptional gene silencing (AGO2), and decapping of mRNA (DCP2). Similar analysis with other APOBEC3 family members revealed a potential link between the localization of APOBEC3G and APOBEC3F to a common ribonucleoprotein complex and P-bodies with potent anti–HIV-1 activity. In addition, we present evidence suggesting that an important role for HIV-1 Vif, which subverts both APOBEC3G and APOBEC3F antiviral function by inducing their degradation, could be to selectively remove these proteins from and/or restrict their localization to P-bodies. Taken together, the results of this study reveal a novel link between innate immunity against retroviruses and P-bodies suggesting that APOBEC3G and APOBEC3F could function in the context of P-bodies to restrict HIV-1 replication.

## Synopsis

Successful replication of viruses and other intracellular pathogens in their respective host cells requires that they overcome a series of replication restrictions or “roadblocks” established by the cell. In the case of HIV-1, the ability of the virus to replicate in human cells is dependent on its ability to neutralize APOBEC3G, a DNA editing enzyme that incorporates into virions and renders them noninfectious. Although a potentially devastating inhibitor of HIV-1 replication, the virus evades APOBEC3G by inducing its degradation during virus assembly. APOBEC3G is also capable of inhibiting the replication of other retroviruses as well as the hepadnavirus hepatitis B, a DNA virus that replicates through an RNA intermediate, suggesting that APOBEC3G may function in cellular defense against a broad range of viral pathogens. Here, Rana and colleagues present their findings that APOBEC3G localizes to specialized compartments in the cytoplasm of mammalian cells known as mRNA processing (P) bodies, which function in the degradation and storage of cellular mRNA. Furthermore, they show that APOBEC3G assembles into a ribonucleoprotein complex with P-body proteins involved in translation, translation suppression, RNA interference, and mRNA decapping. These novel and exciting findings have broad-scale implications for APOBEC3G function and for the role of P-bodies in both cellular defense against viruses and retroviral assembly.

## Introduction

The successful propagation of HIV-1 through the human host has been linked to its ability to subvert and overcome innate cellular defense mechanisms that function by restricting replication of the virus at various points in the life cycle [1]. APOBEC3G is a (deoxy)cytidine deaminase originally discovered as the host restriction factor responsible for limiting the replication of *vif*-deficient HIV-1 [2] and has since been implicated in the restriction of a broad range of exogenous retroviruses [1,3,4], endogenous retroviruses [5,6], and the hepadnavirus hepatitis B [7].

During *vif*-deficient HIV-1 replication, APOBEC3G associates with Gag during viral assembly and is packaged into progeny virions [2,8–11]. Once packaged, APOBEC3G imposes a potent restriction on viral replication in the next target cell through a mechanism that results in genome degradation, incomplete cDNA synthesis, and a detrimentally high mutation rate within the HIV-1 genome [3,10,12–15]. These consequences of APOBEC3G packaging have largely been attributed to deamination of the viral cDNA [3,8–12,15,16]; however, a recent study demonstrated that APOBEC3G remains antiviral in the absence of enzymatic activity [17], suggesting that the capacity of APOBEC3G to restrict HIV-1 replication may extend beyond deamination. Although effective against *vif*-deficient HIV-1, APOBEC3G is neutralized by wild-type HIV-1 through Vif [18–20], which functions in concert with an E3 ubiquitin ligase complex to mediate the polyubiquitination and rapid degradation of APOBEC3G through the proteasome [21–25]. These findings illustrate how HIV-1 has evolved to deactivate an important innate cellular defense mechanism and suggest that therapeutic intervention to disrupt the APOBEC3G-Vif interaction, directly inhibit Vif function, and/or up-regulate APOBEC3G expression could allow the human host to naturally limit the proliferation of HIV-1.

Despite these significant advances in our understanding of APOBEC3G biology, there remained a considerable lack of detail concerning the subcellular context in which APOBEC3G functions. APOBEC3G has been shown to localize throughout the cytoplasm and to concentrate within punctate cytoplasmic bodies [26]. However, the identity or relevance of these cytoplasmic bodies toward the ability of APOBEC3G to restrict HIV-1 replication was unknown. In this report, we show that APOBEC3G cytoplasmic bodies are mRNA processing (P) bodies. P-bodies are found in the cytoplasm of both yeast and mammalian cells and constitute specialized compartments where nontranslating mRNAs accumulate and are subject to degradation or storage [27–29]. In addition to subcellular localization studies, we also present biochemical evidence that APOBEC3G localizes to a ribonucleoprotein (RNP) complex with other P-body proteins which have established functions in cap-dependent translation [30], translation suppression [31,32], RNA interference–mediated post-transcription gene silencing [33–39], and mRNA decapping [27,28,40]. Finally, we present studies that reveal a potential link between the potent anti–HIV-1 activities of APOBEC3G and APOBEC3F with their localization to P-bodies and results suggesting that a primary function of Vif could be to limit the localization of these proteins to P-bodies. Taken together, these findings reveal a novel relationship between innate cellular immunity against retroviruses and P-bodies.

## Results

### APOBEC3G Localizes to mRNA P-Bodies

**Figure 1:**
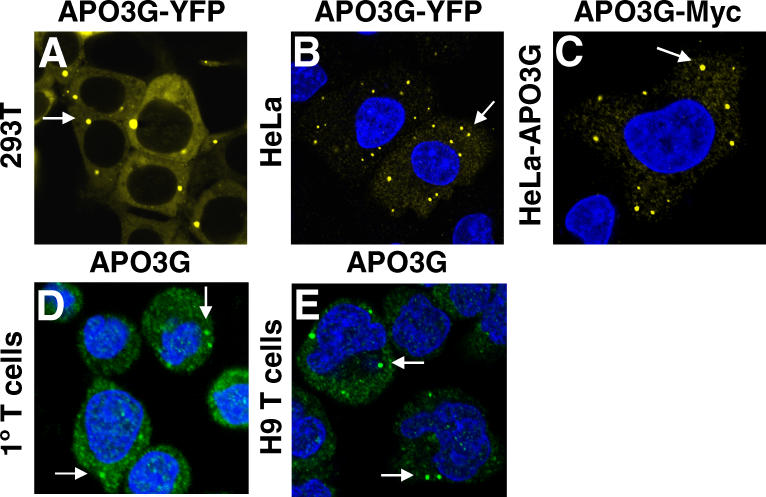
APOBEC3G Localizes to Cytoplasmic Bodies Subcellular localization images of living 293T cells transiently expressing APO3G-YFP (A), HeLa cells transiently expressing APO3G-YFP (B), HeLa-APO3G cells stably expressing APO3G-Myc (C), endogenous APOBEC3G in primary peripheral blood CD4^+^ T cells (D), and endogenous APOBEC3G in H9 T lymphocytes (E). APO3G-YFP was detected by direct YFP fluorescence while APO3G-Myc and endogenous APOBEC3G were detected by indirect immunostaining, using antibodies against the c-Myc epitope and APOBEC3G, respectively. The cells were stained with Hoechst 33258 to visualize nuclei and the imaged were merged digitally. Cell type is noted to the left of each image and arrows point to cytoplasmic bodies.

Recently, we reported that recombinant APOBEC3G localizes throughout the cytoplasm and to discrete cytoplasmic foci of unknown origin, which we referred to as cytoplasmic bodies [26]. This localization pattern was consistently observed when recombinant APOBEC3G was transiently expressed in either 293T cells (e.g., APO3G-YFP in Figure 1A, arrow) or HeLa cells (e.g., APO3G-YFP in Figure 1B, arrow), which renders these naturally permissive cell lines (i.e., cells that lack endogenous APOBEC3G) nonpermissive to *vif*-deficient HIV-1 replication (unpublished data). Similar analysis of HeLa cells that stably express APOBEC3G with a C-terminal c-Myc epitope tag (APO3G-Myc), which also renders these cells nonpermissive to *vif*-deficient HIV-1 replication [41], revealed that stably expressed recombinant APOBEC3G also localized to cytoplasmic bodies (Figure 1C, arrow). Taken together, these findings suggested a possible link between cytoplasmic bodies and the antiretroviral function of APOBEC3G leading us to investigate the subcellular localization of endogenous APOBEC3G in cells that serve as a natural target for HIV-1 infection. Using a rabbit polyclonal antibody directed against the C-terminus of APOBEC3G (see Materials and Methods), we immunolocalized endogenous APOBEC3G in primary CD4^+^ T cells isolated from peripheral blood mononuclear cells following in vitro activation (see Materials and Methods) and in H9 T lymphocytes. In both cases, endogenous APOBEC3G localized throughout the cytoplasm and to cytoplasmic bodies (Figure 1D and 1E, respectively, arrows). Both cell types typically harbored two to ten cytoplasmic bodies per cell, although imaging more than one to three per focal plane was difficult due to the rounded morphology of these cells and their random distribution throughout the cytoplasm. Importantly, these results demonstrated that the localization of APOBEC3G to cytoplasmic bodies was a bona fide property of this protein in T cells.

**Figure 2:**
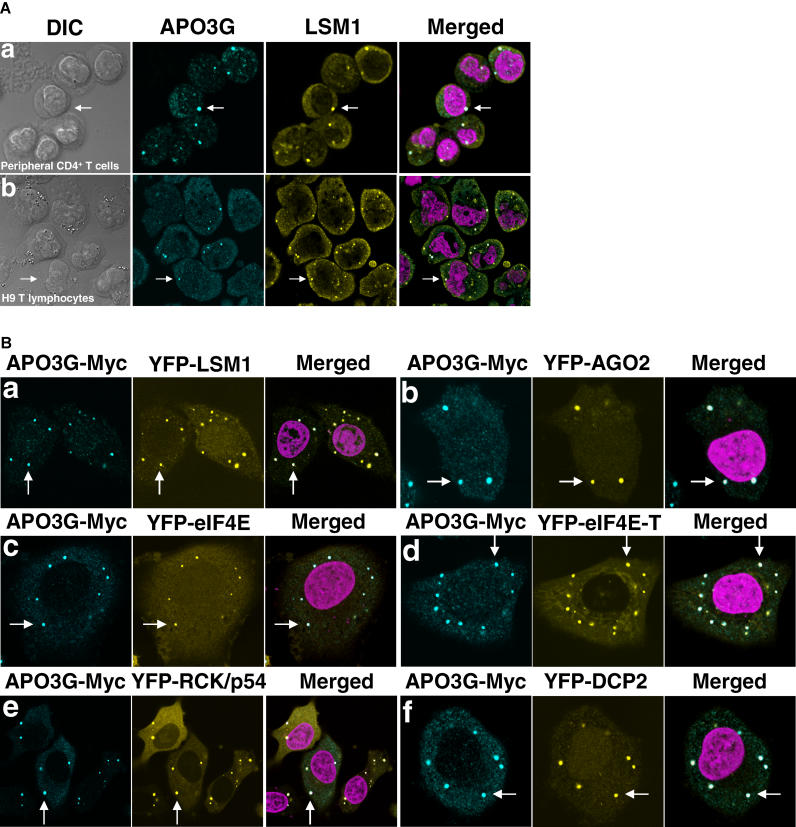
APOBEC3G Cytoplasmic Bodies Are P-Bodies (A) Subcellular localization of APOBEC3G and LSM1 in T cells. Endogenous APOBEC3G and LSM1 were localized in peripheral blood CD4^+^ T cells (a) and H9 T lymphocytes (b) through indirect immunostaining using antibodies against APOBEC3G and LSM1, respectively. The cells were counterstained with Hoechst 33258 to visualize nuclei and the images were digitally merged to highlight regions of colocalization, which appear white in the merged images. A corresponding differential interference contrast (DIC) image is presented to the left and arrows point to cytoplasmic bodies. (B) APOBEC3G colocalizes with P-body proteins at P-bodies. Subcellular localization of APO3G-Myc and YFP-LSM1 (a), YFP-AGO2 (b), YFP-eIF4E (c), YFP-eIF4E-T (d), YFP-RCK/p54 (e), or YFP-DCP2 (f) in HeLa-APO3G cells. APO3G-Myc was detected through indirect immunostaining with an antibody against the c-Myc epitope and YFP was detected by direct fluorescence. The cells were counterstained with Hoechst 33258 to visualize nuclei and the images were digitally merged to highlight regions of colocalization, which appear white in the merged images. Arrows point to P-bodies.

During the course of a series of experiments to characterize the dynamics of APOBEC3G movement in living cells, we observed that cytoplasmic bodies disappeared following a 60-min incubation with cyclohexamide (unpublished data). This finding indicated that cytoplasmic bodies were not static structures but rather were both dynamic and intimately linked to mRNA translation. This dependence on active translation was a strikingly similar feature of proteins that localize to mRNA processing (P) bodies [29,30,42] which function in the degradation and storage on nontranslating mRNAs [27–29,43]. To determine if APOBEC3G cytoplasmic bodies and P-bodies were same structures, APOBEC3G and LSM1, a resident P-body protein [44], were immunolocalized in primary peripheral blood CD4^+^ T cells and H9 T lymphocytes. In both cases, APOBEC3G cytoplasmic bodies overlapped with LSM1-labeled P-bodies (Figure 2A, a and b, respectively, arrows), revealing that APOBEC3G localizes to P-bodies in cells that serve as a natural target for HIV-1 infection. In HeLa-APO3G cells, APO3G-Myc colocalized with endogenous LSM1 at P-bodies (unpublished data) and YFP-tagged versions of the P-body proteins LSM1, AGO2 [35,39], eIF4E [30], eIF4E-T [30], RCK/p54 [27], and DCP2 [28] (Figure 2B, a–f, respectively, arrows), and similar colocalization of these proteins with APO3G-CFP was observed in 293T cells (unpublished data). Taken together, these results clearly demonstrated that APOBEC3G cytoplasmic bodies observed in all of these different cell types were P-bodies.

### Biochemical Analysis of the Interaction between APOBEC3G and P-Body Proteins

**Figure 3:**
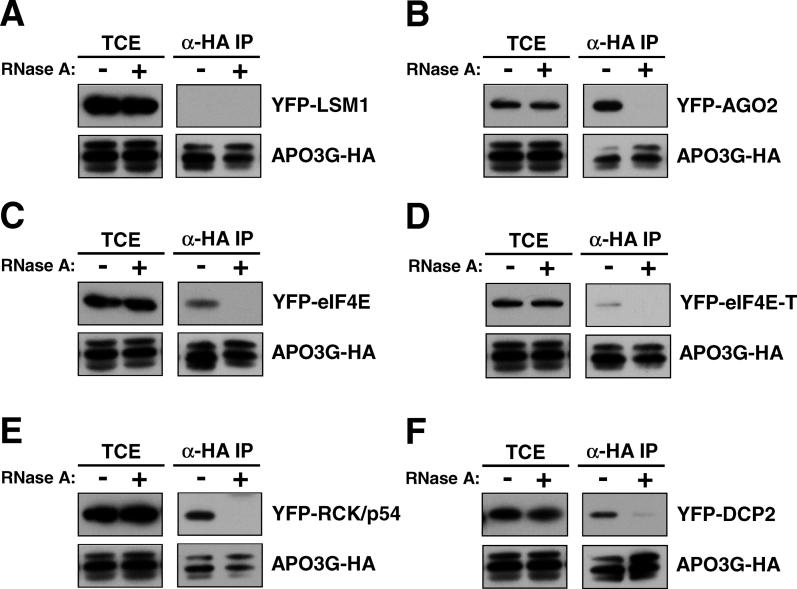
APOBEC3G Interacts with P-Body Proteins through RNA-Dependent Interactions Total cell extracts from 293T cells coexpressing APO3G-HA and YFP-LSM1, YFP-AGO2, YFP-eIF4E, YFP-eIF4E-T, YFP-RCK/p54, or YFP-DCP2 (A–F, respectively) were treated with RNase A (+) or vehicle alone (−) and APO3G-HA was immunoprecipitated (IP) with α-HA agarose (α-HA IP). Total cell extracts (TCE) and α-HA IPs were analyzed by immunoblot, and APO3G-HA was detected with an antibody against the HA epitope, while YFP-tagged P-body proteins were detected with an antibody against GFP.

Next, we investigated whether APOBEC3G simply colocalized with these P-body proteins or if they coexisted within a complex in the cell. Using the YFP-tagged versions of the P-body proteins, we found that YFP-AGO2, YFP-eIF4E, YFP-eIF4E-T, YFP-RCK/p54, and YFP-DCP2 all coimmunoprecipitated with APO3G-HA (Figure 3B–3F, respectively). Interestingly, we could not detect coimmunoprecipitation of YFP-LSM1 (Figure 3A) or endogenous LSM1 (unpublished data) with APO3G-HA, suggesting that despite their colocalization to P-bodies these proteins do not interact in the cell. To determine if these interactions were direct or mediated through cellular RNA, the same extracts from above were also treated with RNase A prior to APO3G-HA immunoprecipitation. In all cases, coimmunoprecipitation of the YFP-tagged P-body proteins with APO3G-HA was significantly reduced by RNase A (Figure 3B–3F), suggesting that the interactions observed between APOBEC3G and AGO2, eIF4E, eIF4E-T, RCK/p54, and DCP2 were mediated through cellular RNA.

### Potent Anti–HIV-1 Activity of APOBEC3 Proteins Correlates to P-Body Localization

Similar to APOBEC3G, APOBEC3F is a potent inhibitor of HIV-1 replication and is targeted by Vif [45–51], while APOBEC3B exhibits only modest anti–HIV-1 activity relative to APOBEC3G and is resistant to Vif due to their inability to interact in the cell [51–55]. As a first step in characterizing the relationship between potent anti–HIV-1 function and the localization of APOBEC3G to P-bodies, we compared the subcellular localization pattern of these APOBEC3 family members.

**Figure 4:**
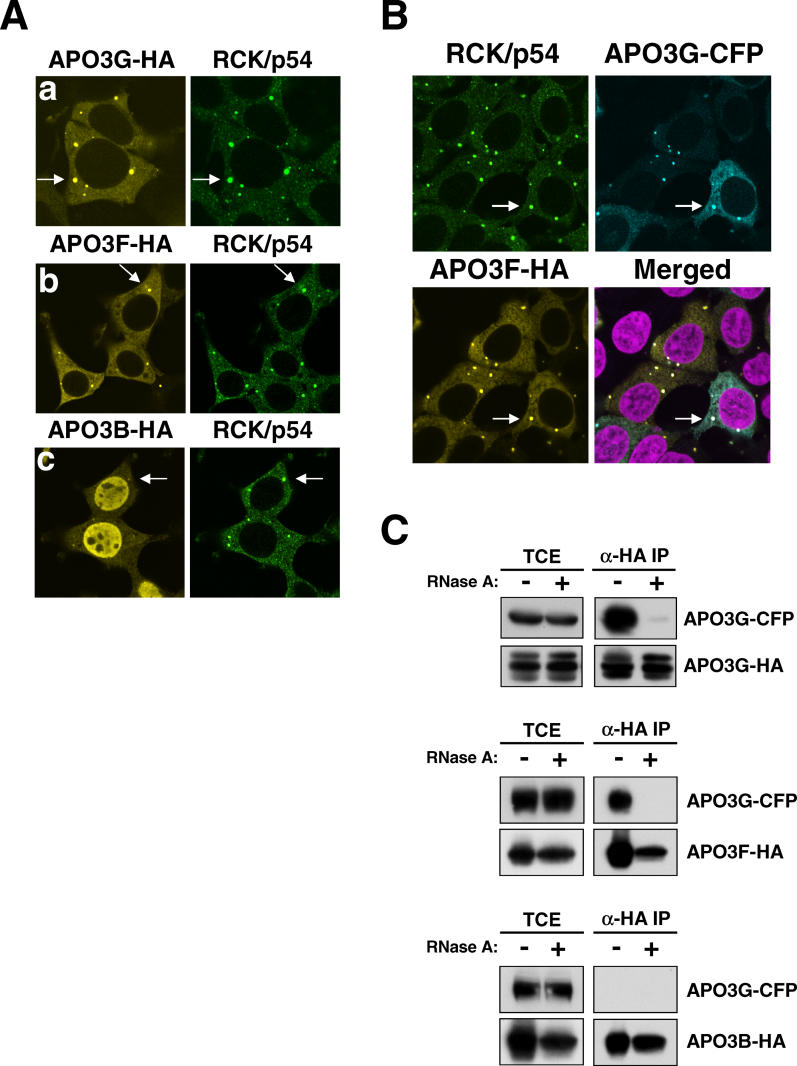
Potent Anti–HIV-1 Activity of APOBEC3 Proteins Correlates to P-Body Localization (A) Subcellular localization of endogenous RCK/p54 and APO3G-HA (a), APO3F-HA (b), or APO3B-HA (c) in 293T cells. Endogenous RCK/p54 was detected through indirect immunostaining with an antibody against RCK/p54 and APOBEC3 proteins with an antibody against the HA epitope. Arrows point to P-bodies. (B) Subcellular localization of endogenous RCK/p54, APO3G-CFP, and APO3F-HA in 293T cells. RCK/p54 was detected through indirect immunostaining with an antibody against RCK/p54, APO3F-HA with an antibody against the HA epitope, and APO3G-CFP was detected by direct CFP fluorescence. The cells were also stained with Hoechst 33258 to visualize nuclei and the images were digitally merged to highlight regions of colocalization, which appear white in the merged image. Arrows point to P-bodies. (C) RNA-dependent interaction of APOBEC3G and APOBEC3F. Total cell extracts from 293T cells coexpressing APO3G-CFP and APO3G-HA (top panel), APO3G-CFP and APO3F-HA (center panel), or APO3G-CFP and APO3B-HA (bottom panel) were treated with RNase A (+) or vehicle alone (−) and HA-tagged proteins were immunoprecipitated (IP) with α-HA agarose (α-HA IP). Total cell extracts (TCE) and α-HA IPs were analyzed by immunoblot and APO3G-CFP was detected using an antibody against GFP while APO3G-HA, APO3F-HA and APO3B-HA were detected using an antibody against the HA epitope.

Similar to APO3G-HA, APO3F-HA localized throughout the cytoplasm and to RCK/p54-labeled P-bodies (Figure 4A, a and b, respectively, arrows). Furthermore, APO3G-CFP and APO3F-HA colocalized at RCK/p54-labeled P-bodies when these proteins were coexpressed in 293T cells (Figure 4B, arrows). These results showed that APOBEC3F localizes to P-bodies and further that the localization of either APOBEC3G or APOBEC3F to P-bodies was not dependent on the other protein. Earlier findings in this study demonstrated that APOBEC3G resides in RNP complexes with other P-body proteins leading us to revisit previous observations that APOBEC3G homo-oligomerizes [26,56,57] and hetero-oligomerizes with APOBEC3F [46] to determine if these interaction were direct or mediated through cellular RNA. To address these possibilities, total cell extracts from 293T cells coexpressing either APO3G-CFP and APO3G-HA or APO3G-CFP and APO3F-HA were treated with RNase A followed by immunoprecipitation of HA-tagged protein using α-HA agarose. RNase A treatment virtually eliminated the coimmunoprecipitation of APO3G-CFP with APO3G-HA and APO3F-HA observed in the control (Figure 4C, top and center, respectively). These results showed that the APOBEC3G-APOBEC3G and APOBEC3G-APOBEC3F interactions previously observed through coimmunoprecipitation studies do not result from direct multimerization but rather these proteins interact through an RNA intermediate.

While APOBEC3G and APOBEC3F coassembled into a common RNP complex and both localized to P-bodies, APO3B-HA was largely restricted to the nucleus of 293T cells (Figure 4A, c) and rarely localized to RCK/p54-labeled P-bodies (less than 10% of cells; 500 cells scored; Figure 4A, c, arrows). Furthermore, a significant interaction between APO3G-CFP and APO3B-HA, relative to that observed for APO3G-CFP and APO3F-HA, was not detected (compare center and bottom panels in Figure 4C). However, considerable overexposure of this immunoblot did eventually reveal a weak and RNA-dependent interaction between APO3G-CFP and APO3B-HA (unpublished data). Taken together with the limited localization of APO3B-HA to P-bodies, these findings suggest that APOBEC3B is not entirely restricted from the RNP complex or P-bodies but rather is not targeted with the same efficiency as either APOBEC3G or APOBEC3F. These intriguing results suggest that the modest anti–HIV-1 activity and resistance to Vif exhibited by APOBEC3B could be linked to its inability to associate with the RNP complex and P-bodies (see Discussion).

### Vif Localizes to P-Bodies in the Presence of APOBEC3G

**Figure 5:**
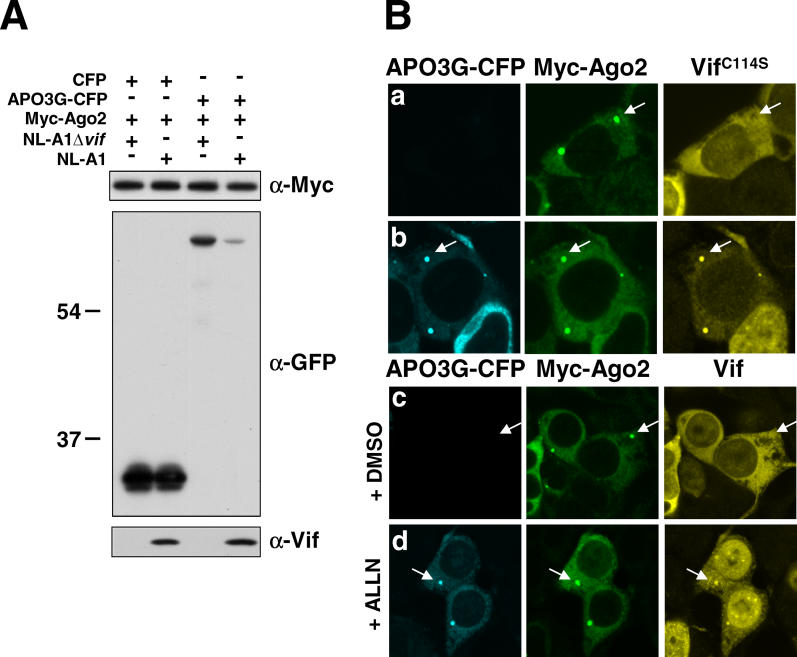
Localization of Vif to P-Bodies Requires APOBEC3G (A) Myc-AGO2 is not sensitive to Vif-mediated degradation in the absence or presence of APO3G-CFP. Total cell extracts from 293T cells expressing CFP or APO3G-CFP with Myc-AGO2 and either NL-A1Δ*vif* or NL-A1 were analyzed by immunoblot with antibodies against CFP (α-GFP), c-Myc (α-Myc) and Vif (α-Vif). Numbers to the left indicate molecular mass in kDa. (B) Subcellular localization images of 293T cells coexpressing a combination of Myc-AGO2 and Vif^C114S^ (a), APO3G-CFP, Myc-AGO2, and Vif^C114S^ (b), or APO3G-CFP, Myc-AGO2, and Vif following a 4-h incubation with the proteasome inhibition ALLN (d) or the equivalent DMSO vehicle control–treated cells (c). APO3G-CFP was detected by direct CFP fluorescence while Myc-AGO2 and Vif protein were detected by indirect immunostaining with antibodies against c-Myc and Vif, respectively. Arrows mark regions of colocalization or lack of colocalization between proteins.

Recently, we reported that coexpression of Vif with APOBEC3G reduced the total cellular levels of APOBEC3G and thus limited its localization to P-bodies (referred to previously as cytoplasmic bodies [26]). In contrast, nonfunctional Vif mutants, such as Vif^C114S^, that continued to interact with APOBEC3G readily colocalized with APOBEC3G at P-bodies [26]. These results showed that Vif was also targeted to P-bodies; however, it was not clear from those studies if the localization of Vif to P-bodies occurred independently or required the presence of APOBEC3G. To investigate these possibilities, we employed Myc-AGO2 as an internal control for P-bodies since this protein was shown to interact with APOBEC3G. The addition of Myc-AGO2 in these experiments also allowed us to examine the specificity of Vif-mediated degradation for APOBEC3G and monitor the consequence(s) of Vif expression on P-bodies. Importantly, in the presence or absence of APO3G-CFP, the steady-state levels of Myc-AGO2 were not affected by the presence of Vif (Figure 5A), demonstrating that Myc-AGO2 was not a target for Vif-mediated degradation. Subcellular localization studies further revealed that in the absence of APO3G-CFP, Vif^C114S^ did not colocalize with Myc-AGO2 at P-bodies (Figure 5B, a, arrows). However, coexpression of APO3G-CFP, Vif^C114S^, and Myc-AGO2 resulted in colocalization of all three proteins at P-bodies (Figure 5B, b, arrows), showing that the localization of Vif^C114S^ to P-bodies was dependent on the interaction with APOBEC3G. When APO3G-CFP, Myc-AGO2, and Vif were all expressed in 293T cells, APO3G-CFP levels were reduced to below detectable levels in more than 90% of the cells that also expressed Myc-AGO2 and Vif. In cells where APO3G-CFP could not be detected, Vif did not colocalize with Myc-AGO2 at P-bodies (Figure 5B, c, arrows). However, inhibiting Vif function through a 4-h incubation with the proteasome inhibitor ALLN not only increased the levels of APO3G-CFP expression but also resulted in the colocalization of APO3G-CFP and Vif at foci that were positive for Myc-AGO2, suggesting that these structures were P-bodies (Figure 5B, d, arrows). Consistent with our previous study [26], proteasome inhibition also resulted in the nuclear localization of Vif (Figure 5B, d). Taken together, these findings demonstrated that the interaction of Vif with APOBEC3G could target Vif to P-bodies but that the proteasome-mediated degradation of APOBEC3G induced by Vif was sufficient to restrict the localization of both proteins to P-bodies. Furthermore, the rapid re-localization of APOBEC3G and Vif to P-bodies following proteasome inhibition suggested that a primary function of Vif could be to restrict the localization of APOBEC3G to P-bodies.

## Discussion

According to our current understanding of APOBEC3G function, this host restriction factor limits the spread of HIV-1 infection, and other retroviruses [1,3,4], by packaging into the virus during assembly in the producer cell. Following infection of the next target cell, APOBEC3G mediates extensive dC-to-dU deamination of the viral cDNA, incomplete cDNA synthesis, and genome degradation [3,10,12–15]. Although effective against *vif*-deficient HIV-1, the potent antiviral activity of APOBEC3G is successfully neutralized by wild-type HIV-1 through Vif [18–20], which functions in concert with an E3 ubiquitin ligase complex to mediate the polyubiquitination and rapid degradation of APOBEC3G through the proteasome [18–20,22,24,58,59].

Despite these significant advances in our understanding of APOBEC3G biology, there remained a considerable lack of detail concerning the subcellular context in which APOBEC3G functions. Cell lines that are permissive (i.e., cells that do not express endogenous APOBEC3G) to *vif*-deficient HIV-1 replication can be rendered nonpermissive through either the transient or stable expression of recombinant APOBEC3G. Previously, we investigated the subcellular localization of recombinant APOBEC3G in these cells lines and reported that the protein localized throughout the cytoplasm and also to punctate cytoplasmic foci [26], which we termed cytoplasmic bodies in reference to the cytoplasmic bodies of another retroviral restriction factor TRIM5α [60]. The fact that these bodies were present under conditions that rendered cells nonpermissive to *vif*-deficient HIV-1 infection suggested to us that these structures could be relevant to the antiviral properties of APOBEC3G.

We began this study by confirming that endogenous APOBEC3G also localizes to cytoplasmic bodies in primary peripheral blood CD4^+^ T cells, establishing that this was a bona fide property of APOBEC3G in cells that serve as a natural target for HIV-1 infection in vivo and leading us to investigate their identity. Our initial studies revealed that APOBEC3G cytoplasmic bodies were distinct from TRIM5α cytoplasmic bodies and further that these bodies did not overlap with endocytic vesicles including early endosomes, late endosomes, or lysosomes (M. J. Wichroski and T. M. Rana, unpublished data). Using translation inhibitors to monitor the kinetics of cytoplasmic body assembly and disassembly, we observed that they disappeared from the cytoplasm within 60 min of cyclohexamide treatment. This dependence on active translation was a remarkably similar property of proteins that localize to mRNA processing (P) bodies, specialized compartments within the cytoplasm of both yeast and mammalian cells where nontranslating mRNAs accumulate and are subject to degradation or storage [27–29,61]. The localization of APOBEC3G to P-bodies raised the possibility that APOBEC3G could interact with other P-body proteins. Biochemical analysis showed APOBEC3G interactions with P-body proteins that function in cap-dependent translation (eIF4E and eIF4E-T [30]), translation suppression (RCK/p54 [31,32]), RNA interference–mediated post-transcriptional gene silencing (AGO2 [35,36,38,39,62]), and decapping of mRNA (DCP2 [27,28,40]). The observation that the interactions between APOBEC3G and these particular P-body proteins were all RNA dependent suggested that APOBEC3G localized to a large multiprotein RNP complex in the cell. Although the significance of the interactions mentioned above toward cellular and/or antiviral APOBEC3G functions remains to be determined, it is of considerable interest that APOBEC3G is associated with cellular machinery that mediates cytoplasmic mRNA processing events. While this manuscript was under review, Beliakova-Bethell et al. [63] reported an intriguing study linking the assembly of the yeast Ty3 retrotransposon virus-like particles with P-bodies. Therefore, it is quite possible that there is a link between the assembly of human retroviruses/retrotransposons and P-bodies.

Our studies on APOBEC3F and APOBEC3B revealed a possible link between the localization of APOBEC3G and APOBEC3F to P-bodies with potent anti–HIV-1 activity. APOBEC3F, which shares approximately 50% sequence identity with APOBEC3G, exhibits potent anti–HIV-1 activity, and is targeted by Vif for proteasome-mediated degradation [45–51], and both proteins are coexpressed in lymphoid cells [55]. Our results showed that APOBEC3F localizes to P-bodies and hetero-oligomerizes with APOBEC3G through an RNA-dependent interaction. On the contrary, APOBEC3B, which shares approximately 59% sequence identity with APOBEC3G, exhibits only modest anti–HIV-1 activity relative to APOBEC3G and is resistant to Vif due to their inability to interact in the cell [51–55]. Interestingly, APOBEC3B rarely localized to P-bodies and was found largely in the nucleus of 293T cells. Furthermore, significant coimmunoprecipitation of APOBEC3B with APOBEC3G was not detected, demonstrating that APOBEC3B does not localize to the same RNP complex shared by APOBEC3G and APOBEC3F. While further studies are necessary to fully assess the role of P-bodies in APOBEC3G antiviral function, these findings provide an interesting link between the potent anti–HIV-1 activities of APOBEC3G and APOBEC3F with their abilities to assemble into an RNP complex and localize to P-bodies. It is also of interest to note that APOBEC3B is not expressed in lymphoid cells [55] and thus would not encounter HIV-1 in vivo, suggesting a likely explanation as to why this protein has not evolved similarly to APOBEC3G and APOBEC3F with respect to anti–HIV-1 activity.

Considering a primary function of HIV-1 Vif is to restrict the incorporation of APOBEC3G into virions, we also determined whether Vif localized to P-bodies. Although the coexpression of Vif and APOBEC3G leads to a significant reduction in APOBEC3G levels, it is possible to detect cells where APOBEC3G and Vif are visible in the same cell [26]. In these cases, the remaining APOBEC3G rarely localized to P-bodies; however, in cases where P-body localization could be detected we noticed that Vif localized to these P-bodies as well. This observation suggested that Vif could localize to P-bodies in the presence of APOBEC3G but that the reduction in APOBEC3G levels mediated by Vif also restricted its own localization to P-bodies. This hypothesis was confirmed when it was shown that proteasome inhibition or coexpression of APOBEC3G with the Vif ^C114S^ mutant, a nonfunctional Vif mutant that continues to interact with APOBEC3G [26], led to complete colocalization of Vif and APOBEC3G at P-bodies. Interestingly, Vif did not localize to P-bodies in the absence of APOBEC3G, showing that the APOBEC3G-Vif interaction was responsible for targeting Vif to P-bodies. The finding that Vif-mediated degradation restricted the localization of both proteins to P-bodies suggests that an important role for Vif could be to selectively remove APOBEC3G from and/or prevent the association of APOBEC3G with P-bodies.

One of the more intriguing aspects of APOBEC3G biology is that it targets a broad range of both exogenous [1,3,4] and endogenous [5–7] retroviruses and can also limit the production of infectious hepatitis B virus [7,64–67], a DNA virus the replicates through an RNA intermediate. These findings suggest that APOBEC3G and other members of the APOBEC3 family of proteins may constitute a broad antiviral defense mechanism. This hypothesis is supported by recent findings demonstrating that the expression of APOBEC3G and other APOBEC3 family members is induced by interferon-α in primary monocyte-derived macrophages [68] and primary hepatocytes [69], suggesting that these proteins could be up-regulated in vivo as part of the innate immune response to viral infection. The findings that APOBEC3G and APOBEC3F are associated with mRNA processing machinery provide an important clue as to how these proteins may have evolved to exploit a common theme to protect the cell from a broad range of foreign genetic elements. Future studies would reveal the role of P bodies in the assembly of human retroviruses and retrotransposons.

## Materials and Methods

### Expression vectors and antibodies.

APOBEC3G expression vectors pAPO3G-CFP, pAPO3G-YFP, and pAPO3G-HA were described previously [26]. Expression vectors pAPO3F-HA and pAPO3B-HA, which express APOBEC3F and APOBEC3B, respectively, with a C-terminal HA tag, were generous gifts of Dr. Bryan Cullen [54]. The pMyc-AGO2 expression vector, which expresses AGO2 with an N-terminal c-Myc epitope tag, was a generous gift of Dr. Gregory Hannon [70]. Vectors for the expression of YFP-tagged versions of LSM1 were engineered by PCR amplification of their corresponding coding sequences from 293T cDNA followed by cloning into the BglII and SalI sites of pEYFP-C1 (BD Biosciences). The HIV-1 subgenomic proviral vectors pNL-A1, pNL-A1C1, which harbors the Vif^C114S^ mutant, and pNL-A1Δvif were generous gifts of Dr. Klaus Strebel [41]. Antibodies used in this study include mouse monoclonal antibodies α-GFP (BD Biosciences, San Diego, California, United States), α-p24 Gag (Advance Biotechnologies, Columbia, Maryland, United States), α-GST (Santa Cruz Biotechnology, Santa Cruz, California, United States), α-HA (Santa Cruz Biotechnology), and α-Vif (ImmunoDiagnostics, Woburn, Massachusetts, United States), rabbit polyclonal antibodies α-HA (Santa Cruz Biotechnology) and α-DDX6 (RCK/p54; Bethyl Laboratories, Montgomery, Texas, United States), and chicken polyclonal antibody α-LSM1 (GenWay Biotech, San Diego, California, United States). All chemicals were purchased from Sigma (St. Louis, Missouri, United States) unless otherwise indicated.

### Manipulation of mammalian cells.

Primary CD4^+^ T cells were isolated (Dynal Biotech ASA, Oslo, Norway) from PHA/IL-2 activated human peripheral blood mononuclear cells cultured in RPMI 1640 medium (Invitrogen, Carlsbad, California, United States) supplemented with 10% fetal bovine serum, 100 units/ml penicillin, and 100 μg/ml streptomycin. The H9 lymphoid T cell line (ATCC [American Type Culture Collection], Manassas, Virginia, United States) was cultured in RPMI 1640 medium modified as above. The human 293T embryonic kidney and HeLa cervical carcinoma cell lines were maintained in a humidified incubator (5% CO_2_) at 37 °C in Dulbecco's modified Eagle's medium (Invitrogen) also modified as above. The HeLa-APOBEC3G cell line (referred to here as HeLa-APO3G), which stably expresses APOBEC3G with C-terminal c-Myc epitope tag (APO3G-Myc), was obtained through the National Institutes of Health AIDS Research and Reference Reagent Program, Division of AIDS, National Institute of Allergy and Infectious Diseases, from Drs. Klaus Strebel and Eri Miyagi [41]. 293T and HeLa cells were transfected with Qiagen-purified plasmid DNA (Qiagen, Valencia, California, United States) using LipofectAMINE 2000 (Invitrogen) as previously described [26].

### Immunoprecipitation and immunoblot analysis.

For immunoprecipitation, total cell extracts were prepared using Mammalian Protein Extraction Reagent (M-PER; Pierce, Rockford, Illinois, United States) supplemented with 0.5% (v/v) Triton-X 100 (Pierce), 150 mM NaCl, 5 mM EDTA, and a 1:100 (v/v) dilution of a protease inhibitor cocktail for mammalian tissue. Extracts were clarified by centrifugation and protein concentration was determined by D_c_ protein assay (Bio-Rad, Hercules, California, United States). HA and CFP tagged proteins were precipitated by overnight incubation with either α-HA or α-GFP rabbit polyclonal antibodies directly conjugated to agarose beads (Santa Cruz Biotechnology). Samples were washed four times (15 min for each wash) in lysis buffer and eluted by boiling for 2 min at 100 °C in SDS-PAGE sample buffer (50 mM Tris-HCl [pH 6.8], 100 mM dithiothreitol, 2% [w/v] SDS, 0.1% [w/v] bromophenol blue, 10% [v/v] glycerol). SDS-PAGE separation and immunoblot analysis of protein were performed as previously described [26].

### Immunolocalization.

For immunolocalization, 293T and HeLa cells were seeded onto glass bottom micro-well dishes coated with poly-d-lysine (MatTek Corporation, Ashland, Massachusetts, United States). Primary peripheral blood CD4^+^ T cells and H9 T lymphocytes were attached to cover slips using Cell-Tak cell and tissue adhesive (BD Biosciences) according to the manufacturer's instructions. Cells were fixed for 20 min with 3.7% (v/v) paraformaldehyde (Electron Microscopy Sciences, Hatfield, Pennsylvania, United States) in PBS for 20 min followed by permeabilization with 0.25% (v/v) TX-100 (Pierce) for 5 min. Samples were next washed three times (5 min for each wash) in PBS containing 0.1% (v/v) TX-100 (PBST). Samples were blocked for 30 min in PBST containing 2% (w/v) bovine serum albumin (PBST/BSA). All primary and secondary antibodies were diluted in PBST/BSA and secondary antibodies were directly conjugated to Alexa Fluor dyes (Molecular Probes, Eugene, Oregon, United States). In certain cases, samples were counterstained with Hoechst 33258 to visualize the nuclei. Samples were visualized with a Leica confocal imaging spectrophotometer system (TCS-SP2) (Exton, Pennsylvania, United States) attached to a Leica DMIRE inverted fluorescence microscope and equipped with an argon laser (458-, 476-, 488-, 514-nm lines), two HeNe lasers (543-, 633-nm lines), an acousto-optic tunable filter (AOTF) to attenuate individual visible laser lines, and a tunable acousto-optical beam splitter (AOBS). All images were acquired from a ×63, 1.32 NA oil immersion objective with variable digital magnification ranging from ×4 to ×8, and image analysis was performed using Leica Confocal Software (LCS) and Adobe Photoshop 7.0 (Adobe Systems, Mountain View, California, United States).

## Supporting Information

### Accession Numbers

The GenBank accession numbers (http://www.ncbi.nlm.nih.gov/Genbank) for the genes and gene products mentioned in this paper are YFP-tagged versions of LSM1 (NM_014462), AGO2 (NM_012154), eIF4E (NM_001968), eIF4E-T (NM_019843), RCK/p54 (NM_004397), and DCP2 (NM_152624).

## Abbreviations

P-body: processing body
RNP: ribonucleoprotein
